# Exploring the drivers of digital transformation in Chinese port and shipping enterprises: A machine learning approach

**DOI:** 10.1371/journal.pone.0322872

**Published:** 2025-05-05

**Authors:** Jiahui Jin, Yongchun Guo

**Affiliations:** 1 School of Maritime Economics and Management, Dalian Maritime University, Dalian, Liaoning, China; 2 School of Public Finance and Taxation, Dongbei University of Finance and Economics, Dalian, Liaoning, China; Tongji University School of Economics and Management, CHINA

## Abstract

With the transition to a global green low‐carbon economy, the urgency for digital transformation in the port and shipping industry has become increasingly prominent in making enterprises more efficient and sustainable. This study focuses on how Chinese port and shipping enterprises, which are key carriers for global containerized trade, can attain digital transformation as a means to tackle environmental challenges and improve competitiveness. Using a representative sample of 83 A-share-listed companies (2008–2023) and employing several modeling techniques, such as Ridge regression, LightGBM, and XGBoost, we investigate a data-driven approach with the support of the Technology–Organization–Environment (TOE) framework. We find that nonlinear models (LightGBM, XGBoost) outperform linear models and emphasize the importance of a supportive environment for green finance. We further perform a number of sensitivity and robustness checks toensure the validity of our findings. These insights provide actionable guidance for policymakers and industry leaders seeking to harmonize digital innovations with green development.

## 1. Introduction

As globalization and informatization are inextricably intertwined with each other, port and shipping enterprises are facing unprecedented challenges and opportunities. The recovery of international trade and the increasing complexity of global supply chains demand that port and shipping enterprises improve operational efficiency and service capabilities, while placing greater emphasis on corporate social responsibility in environmental protection and sustainable development. As a key player in global maritime trade, China boasts the largest fleet in the world, accounting for 30.1% of global shipping volume of foreign trade, with the port container throughput reaching 310 million TEUs and seven major ports ranking in the top ten worldwide (China Port Operation Analysis Report, 2024). Research indicates that digital transformation provides new opportunities for the green development of port and shipping enterprises, enhancing their market competitiveness and making significant contributions to achieving global carbon reduction targets [1]. The widespread application of digital technologies, such as big data, the Internet of Things (IoT), and blockchain, not only supports the optimized management of resources but also promotes the formation of green supply chains, making the operations of port and shipping enterprises more efficient [2,3]. Digital transformation has become a necessary means to enhance the competitiveness of Chinese port and shipping enterprises and optimize operational efficiency.

Meanwhile, with the acceleration of the global green and low-carbon transition, the role of port and shipping enterprises in fulfilling their social responsibility has become increasingly important. Digital transformation not only enhances the operational efficiency of enterprises but also contributes to the achievement of environmental protection and resource conservation goals. In this context, green development has become an important guiding principle in national policies, and the digital transformation of port and shipping enterprises is a concrete practice of this concept. By utilizing technological means to optimize business processes and improve resource management efficiency, port and shipping enterprises can reduce operational costs while promoting environmental protection and creating social value on a broader level. Therefore, constructing a theoretical framework of green development driven by the digital transformation of port and shipping enterprises and exploring its implementation paths and strategies has profound academic and practical significance.

This paper aims to analyze the driving factors behind the digital transformation of Chinese port and shipping enterprises based on the TOE (Technology-Organization-Environment) framework, exploring how digital transformation promotes green development within enterprises and deriving key strategic recommendations. The study not only provides new theoretical perspectives for the academic community but also offers practical guidance for port and shipping enterprises to better fulfill their social responsibility and promote green and sustainable development while facing the challenges of digital transformation. The main contributions of this paper are three folds. First, it reveals the importance of digital transformation in port and shipping enterprises, clarifying its role in enhancing corporate competitiveness and driving green development. Second, based on the TOE framework, it systematically analyzes the driving factors behind digital transformation in port and shipping enterprises, filling the gap in existing research regarding the factors influencing digital transformation. Finally, it offers strategic recommendations for the digital transformation of port and shipping enterprises, providing practical guidance for achieving green and sustainable development.

This paper is divided into five parts: The first part is the introduction, which presents the research background, motivation, and significance. The second part is the literature review, which organizes relevant theories and research findings. The third part outlines the research methods, detailing the research design and data sources. The fourth part presents the empirical analysis, showcasing the data analysis results and key findings. The fifth part provides conclusions and recommendations, summarizing the research conclusions and proposing corresponding strategies.

## 2. Literature review

In recent literature regarding digital transformation in the maritime sector, three interconnected areas have received particular attention:

- Driving Factors:

The digital transformation of port and shipping enterprises is driven by various factors, including policies, market demands, and technological innovations. Firstly, policies, regulations, and international agreements, such as carbon taxes and the “dual carbon” goals, provide a strong external impetus for enterprises, compelling them to adopt green technologies to meet increasingly stringent regulatory requirements. Research indicates that carbon tax policies and government subsidies have effectively promoted the application of low-carbon technologies [4–10]. Secondly, intensified market competition and growing consumer demand for environmentally friendly products have prompted enterprises to enhance their green management levels through digital means to gain a competitive advantage [11,12]. Additionally, managers’ emphasis on green development and the enhancement of enterprises’ dynamic capabilities have played significant roles in driving the digital transformation process [13–15]. Finally, the widespread application of big data analytics, resource integration, and blockchain technology has increased enterprises’ adaptability and flexibility in managing green supply chains, enabling them to effectively reduce carbon emissions through real-time data monitoring and cargo tracking [16–18].

- Transition Models and Technology Adoption:

The rising adoption of advanced technologies (due to the advances in the Internet of Things (IoT), Artificial Intelligence (AI), blockchain, and big data analytics) has facilitated the creation of smart ports and its efficiencies together [4,11]. While game-theoretic frameworks and historical perspectives are also used to portray the development of digital transformation in maritime context [19], more nuanced analyses find their way through game-theoretic frameworks and historical perspectives. Moreover, recent studies highlight the interconnected nature of factors which not only drive or hinder digitalization, but also inform its impact on port communities [20].

- Green Development Outcomes:

Port managers hear about digital transformation more and more as a leading route to bolster the sustainability ambitions of their Port Operations. The use of digitalization allows ports to accomplish better utilization of resources and a reduction of emissions contributing sustainable development [12,21,22] Research by Su et al. (2024) that has explicitly reported on determinants of success for the transformation of green ports through digital technologies, supporting the notion that digital innovation in many cases correlates very closely with environmental performance [23].

Taken together, these studies highlight the underscores the significance of a multidimensional approach—one that incorporates technological, organizational, and environmental elements—in providing insights into the digital transformation of port and shipping firms. The Technology–Organization–Environment (TOE) framework is well suited for this as it allows for an important analysis of the interplay between internal capabilities and external pressures shaping digital strategies [24,25].Together with the incorporation of new views, e.g., historical analysis and game theoretic modeling,these already add up to an undisputed contribution to our understanding and insights as to the challenges and opportunities that this transformation holds [19].

Past studies have provided a foundational framework for the study of digital transformation in the maritime industry, revealing its key drivers and consequences, whilst recent studies have enriched our understanding of the relationship between digitalization and green development. Recent literature in this area not only reveals the technology innovation enabling this transformation, but also the strategic need for sustainable integration of sustainably integrating digital opportunities into practice.

## 3. Theoretical foundation

With the acceleration of globalization and the transformation of trade patterns, port and shipping enterprises, as key nodes in the international logistics chain, are facing a complex and dynamic external environment as well as increasingly fierce market competition. Digital transformation is not only a essential path for port and shipping enterprises to improve operational efficiency, optimize resource allocation, and enhance competitiveness, but also the core driving force for achieving green development and promoting sustainable growth. This study will conduct an in-depth analysis of the internal and external factors influencing the digital transformation of port and shipping enterprises using the Technology-Organization-Environment (TOE) framework, and explore its application in the path of green development.

### 3.1. Definition of port and shipping enterprises

Port and shipping enterprises refer to comprehensive enterprises engaged in various business activities within port operations, shipping services, shipbuilding, and maintenance. These enterprises primarily include port facility construction and management, ship operations, cargo transportation, logistics services, as well as the design, construction, and maintenance of ships [26]. Port and shipping enterprises ensure the efficient flow of international trade and logistics through the provision of shipping and port services. Additionally, some of these enterprises are also involved in the ship production and technological innovation, thus forming a complete industrial chain. these enterprises not only handle infrastructure operations and transportation management but also play a crucial role in shipbuilding, the advancement of maritime technology, and other related areas, exerting a strategic influence on the functioning of the global economy [27].

In this study, “port and shipping enterprises” refers to the complex, interconnected, and collaboratively developed enterprise ecosystems formed through multidimensional activities in port management, shipping services, and shipbuilding within the globalized economic system.

### 3.2. Technology-Organization-Environment (TOE) framework

The TOE framework is particularly suited for our study as it captures the diverse influences that shape the digital transformation. Technological factors (emerging tools, irrespective of IoT, AI, and advanced protocols/blockchain) enable advanced data integration and operational integration for efficiency [24]. Organizational factors, such as the financial health and innovation culture an enterprise has, help to determine an enterprise’s capacity to adopt digital solutions. External drivers for transformation are environmental factors such as government policies and pressures in the market. Our choice of indicators (Table 1) was informed by recent international research and specifically aims to depict this complex relationship [28,29].

**Table 1:** Relevant Model Indicators and Explanations.

Dimension	Indicator	Explanation
**Technology**	Degree of Digital Transformation A	According to [34], the frequency of 76 digitalization-related terms was analyzed across five dimensions: artificial intelligence technology, big data technology, cloud computing technology, blockchain technology, and the use of digital technologies.
	Degree of Digital Transformation B	According to [35], the frequency of 99 digital-related terms was analyzed across four dimensions: digital technology applications, internet business models, intelligent manufacturing, and modern information systems.
	Degree of Digital Transformation C	According to [36], the frequency of 139 digitization-related terms was analyzed across categories such as technology classification, organizational empowerment, and digital applications.
	Digital Application - Business Innovation	Evaluates the application of digital technologies in driving business innovation, reflecting how enterprises use digital technology to innovate business models, products, or services to meet market demands.
	Digital Inclusive Finance Comprehensive Index	Reflects the company’s application of digital technologies in the field of inclusive finance, indicating how the company uses technology to expand the accessibility and reach of financial services, particularly for low-income groups.
	Digital Technology Application - AS Score	Measures the company’s comprehensive evaluation in digital technology application, assessing how extensively the company utilizes various technologies (such as cloud computing, big data, and AI).
	Total Number of Green Patent Applications	Represents the company’s innovative performance in the field of environmental protection technologies, as indicated by the number of patent applications related to green technologies and sustainable development.
	R&D Expenditure/ Total Assets	Measures the proportion of the company’s resources allocated to research and development as a percentage of its total assets, showcasing the company’s commitment to technological innovation.
	Proportion of R&D Investment to Revenue (%)	Reflects the proportion of the company’s revenue allocated to research and development, underscoring the company’s focus on technological innovation and R&D.
	Ln (1 + Number of Patents)	The natural logarithm of the number of patents, reflecting the company’s accumulation in technological innovation and R&D. Patent numbers are typically an important indicator of innovation capability.
**Organization**	Net Profit/ Total Assets	Measures the company’s ability to generate net profit from its assets, reflecting the the efficiency of resource allocation in operations.
	Natural Logarithm of Total Assets at Year-End	Calculates the natural logarithm of the company’s total assets at year-end, which is typically used to measure company size. A larger asset base often indicates stronger market competitiveness and resource allocation capability.
	Natural Logarithm of Company Listing Duration (Observation Year - IPO Year)	Reflects the number of years since the company’s listing, with the natural logarithm transformation to mitigate bias introduced by longer durations, thereby to better represent the company’s maturity and time of development in the market.
	Number of Independent Directors	Measures the number of independent directors in the company’s governance structure. Independent directors typically provide objectivity and independence in decision-making and supervision, helping to improve the transparency and fairness of corporate governance.
	Proportion of R&D Investment to Revenue (%)	Reflects the proportion of the company’s revenue allocated to research and development, typically indicating the company’s investment in technological innovation.
**Environment**	E Score	Environmental (E) score, assessing the company’s performance in environmental protection and sustainability, including responsibilities in areas such as energy use, emissions, and resource utilization.
	G Score	Governance (G) score, measuring the company’s performance in corporate governance, including transparency, board structure, management accountability, and other factors.
	Huazheng ESG Score	ESG (Environmental, Social, Governance) composite score assess the company’s performance in environmental, social responsibility, and corporate governance, as provided by Huazheng.
	Annual Average	Measures the average annual performance of the company, which may be an average score of multiple indicators, typically used to reflect the company’s overall environmental or business performance during a specific time period.
	Ln (1 + Number of Patents)	The natural logarithm of the number of patents, reflecting the company’s achievements in technological innovation. Although it is a technological indicator, it is also influenced by external factors such as legal protection, market demand, and therefore is partly categorized under the environmental dimension.

Technological Factors: The digital transformation of port and shipping enterprises depends heavily on modern information technologies and infrastructure. The widespread application of technologies such as the Internet of Things (IoT), big data, Artificial Intelligence (AI), and blockchain can enhance the synergy of information flows, logistics, and financial flows, thereby improving operational efficiency [28]. In particular, under the influence of technologies such as port automation, intelligent logistics, and autonomous transportation vehicles, port and shipping enterprises can not only enhance production efficiency and reduce costs but also achieve green and low-carbon development.

Organizational Factors: The internal resource allocation, technological absorption capacity, management innovation, and top-level support are critical to the success of digital transformation. The innovation culture of the enterprise, the strategic vision of its leadership, and the technological adaptability of its employees all influence the process and outcomes of the transformation [25]. For example, port and shipping enterprises need to reform organizational structures and management processes to deepen the application of information technologies, thus optimizing complex business processes.

Environmental Factors: In the global supply chain, port and shipping enterprises cannot ignore the impact of external market demand, government policies, and industry development trends. Government policies such as green development initiatives, carbon emission control, and environmental protection regulations will directly push enterprises to accelerate their digital and green transformation [30,31]. At the same time, the competitiveness of global trade, customer demand for green transportation services, and environmental sustainability pressures all serve as external drivers for port and shipping enterprises to adopt digital transformation strategies.

### 3.3. Transaction cost theory

Transaction Cost Theory (Williamson, 1986) provides an alternative perspective for analyzing the digital transformation of port and shipping enterprises. Digital transformation helps significantly reduce transaction costs for port and shipping enterprises in areas such as information search, contract negotiation, transaction execution, and post-transaction supervision. In traditional port and shipping businesses, issues such as information asymmetry, delays in logistics information, and inefficiencies in human resource management often lead to high transaction costs [32]. The application of digital platforms can optimize the flow of information through real-time data sharing, intelligent scheduling, and supply chain visualization, thereby reducing transaction costs [33]. Furthermore, port and shipping enterprises can implement automated scheduling and transportation process monitoring through intelligent systems, thereby reducing human error and resource waste, and enhancing operational efficiency.

## 4. Data and methods

### 4.1. Data collection and processing

This paper aims to explore the key factors influencing the digital transformation of publicly listed port and shipping enterprises in China. To ensure the scientific rigor of the research and the reliability of the data, we selected 83 A-share listed port and shipping enterprises in China as the research subjects based on such criteria as industry classification, the main business of the enterprises, and certain financial and operational indicators. These enterprises are highly representative at the forefront of digital transformation and can adequately reflect the overall development trends of the port and shipping industry in China. Furthermore, the data of listed companies are publicly available, transparent, and disclosed as per standard procedure, making it easier to access and verify, while also offering high representativeness and comparability. The data primarily come from the Wind database, annual reports of the enterprises, and relevant industry reports, covering a 15-year period from 2008 to 2023. The selected variables include organizational factors, technological factors, and environmental factors. These indicators comprehensively reflect the financial health, digitalization progress, and the impact of the green financial environment on the digital transformation of enterprises, providing a solid foundation for the research. The specific indicators are shown in Table 1.

During the data processing phase, strict screening was conducted for missing and outlier values in the data. Variables with more than 50% missing values were discarded to ensure the integrity of the data and the reliability of the analysis. For the remaining missing values, the median of each column was used for imputation. This approach preserves the central tendency of the data while avoiding the influence of extreme values on the analysis results. For outliers, the 3σ (Three-Sigma) rule was applied to ensure the validity of the data distribution and prevent outliers from having an excessive impact on model fitting. In addition, to further improve the quality and applicability of the data, standardization was performed. Standardization eliminates the impact of different units of measurement across variables, ensuring that each variable contributes more equally to the model. This process is crucial for the subsequent model training and interpretation of the results.

### 4.2. Research methods

#### 4.2.1. *Multi-model algorithms.*

We employed several different modeling techniques, ranging from Multiple Linear Regression, LASSO, and Ridge Regression, to LightGBM and XGBoost, in order to capture both linear and non-linear relationships across our indicators. In addition to 5-fold cross-validation, we performed 10-fold cross-validation and sensitivity analyses on hyperparameters of the model. The supplementary tests (see Table 2) enhance the robustness and generalizability of our results.

**Table 2:** Comparison Table of Digital Transformation Analysis Models for Ports and Maritime Enterprises.

Multiple Linear Regression	Description	Applicable Scenarios
	A classic statistical method applicable for analyzing the linear relationship between the dependent variable and multiple independent variables. The model helps quantitatively assess the impact of technological, organizational, and environmental factors on digital transformation, revealing the strength of driving factors.	Quantitative analysis of the impact of technical, organizational, and environmental factors on the digital transformation of port and maritime enterprises, describing the direct effects of driving factors on the extent of transformation.
Least Absolute Shrinkage and Selection Operator	By introducing L1 regularization for shrinkage and selection of feature coefficients, it is suitable for feature selection in high-dimensional data. LASSO regression can automatically screen out the factors that have the greatest impact on digital transformation, solving the problem of numerous variables and difficulty in identifying core variables.	Feature selection, identifying key driving factors in the digital transformation of port and maritime enterprises, addressing the issue of core variable identification in high-dimensional data.
Ridge Regression	Introducing L2 regularization effectively addresses the problem of multicollinearity, maintaining the stability of regression coefficients and ensuring the reliability of regression results, especially suitable for situations where technological and organizational factors are highly correlated.	Applicable to the problem of multicollinearity, analyzing the technical and organizational factors in the digital transformation of port and maritime enterprises, ensuring the stability and reliability of regression results.
Light Gradient Boosting Machine	Based on the gradient boosting framework, it has high computational efficiency and powerful nonlinear modeling capabilities. Its fast training speed and high accuracy are particularly suitable for processing large-scale, high-dimensional data.	Handling large-scale, high-dimensional data to reveal the complex interactions between technological, organizational, and environmental factors in the digital transformation of port and maritime enterprises, and performing feature importance analysis.
Extreme Gradient Boosting	An efficient gradient boosting method widely applied in modeling complex multivariate problems. Its superior predictive performance and resistance to overfitting, especially when dealing with nonlinear relationships and data imbalance, are outstanding.	Precisely modeling the driving factors of the digital transformation of port and maritime enterprises, analyzing the relative importance and mechanism of action of each factor, especially suitable for handling nonlinear and data imbalance issues.

In addition, to evaluate the stability and generalization performance of the models, this study uses 5-fold cross-validation. This approach divides the data set into five subsets, where each subset is used as the validation set in turn, while the remaining subsets serve as the training set. The training and evaluation process is repeated for each fold, allowing for the calculation of the model’s average performance metrics across all subsets.

## 5. Empirical results

### 5.1. Model evaluation and comparison

By comparing the evaluation metrics of each model, we can gain a comprehensive understanding of how well different models identify and analyze the impact of financial indicators, digitalization indicators, and green finance indicators on the digital transformation of port and shipping enterprises. This study uses Python 3.11 to first evaluate the metrics of each model. The evaluation results show that the LightGBM and XGBoost models perform exceptionally well across all evaluation metrics, especially in terms of the coefficient of determination (R²), with both models approaching 1. This suggests that these models are highly effective in explaining the variability in the dependent variable.

The summary of the evaluation metrics for each model is as Table 3:

**Table 3:** Summary of Evaluation Metrics for Each Model.

Model	MSE	RMSE	MAE	R^2^
Multiple Linear Regression	2.1255 × 10^27^	4.6103 × 10^13^	1.8516 × 10^12^	−2.0995 × 10^23^
LASSO Regression	1513.28	38.90	30.36	0.8505
Ridge Regression	1487.46	38.57	29.65	0.8531
XGBoost	452.14	21.26	11.86	0.9553
LightGBM	421.76	20.54	12.15	0.9583

### 5.2. Cross-validation results

To evaluate the stability and generalizability of the models, we used both (5-fold) and (10-fold) cross-validation. The results are provided in Table 4; for lightgbm and XGBoost models the mean R² scores were 0.9387 and 0.9353, accordingly. Further sensitivity analyses that included varying regularisation parameters across a wide spectrum showed stable model performance, thereby reinforcing our confidence in the robustness of our results. Full test results are available in the Table 4.

**Table 4:** Cross-Validation R² Scores of Each Model.

Model	Cross-Validation R²
Multiple Linear Regression	−1.0888 × 10^23^
LASSO Regression	0.8218
Ridge Regression	0.8083
XGBoost Regression	0.9353
LightGBM Regression	0.9387

As shown in Table 4, the multiple linear regression model exhibits extremely poor performance in the cross-validation, with an average R² score of −1.0888 × 10²³, further validating its unsuitability for this study. Both LASSO regression and Ridge regression exhibit comparatively stable performance in the cross-validation, with average R² scores of 0.8218 and 0.8083, respectively. This indicates that these two models maintain consistency in performance across datasets. The XGBoost and LightGBM models exhibit excellent performance in the cross-validation, with average R² scores of 0.9353 and 0.9387, respectively. This further validates their superiority in model stability and generalization.

### 5.3. Feature importance analysis

The purpose of the feature importance analysis is to identify factors significantly impacting on the digital transformation of enterprises, based on the results from various models. In this study, we used both the LightGBM and XGBoost models, which evaluate feature contributions model predictions based on different principles.

**Fig 1:**
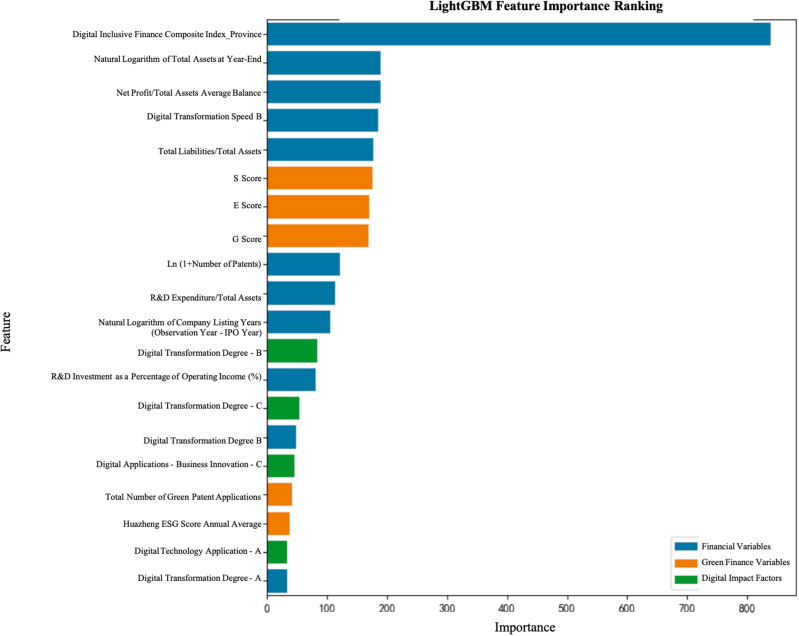
Feature Importance of the LightGBM Model.

**Fig 2:**
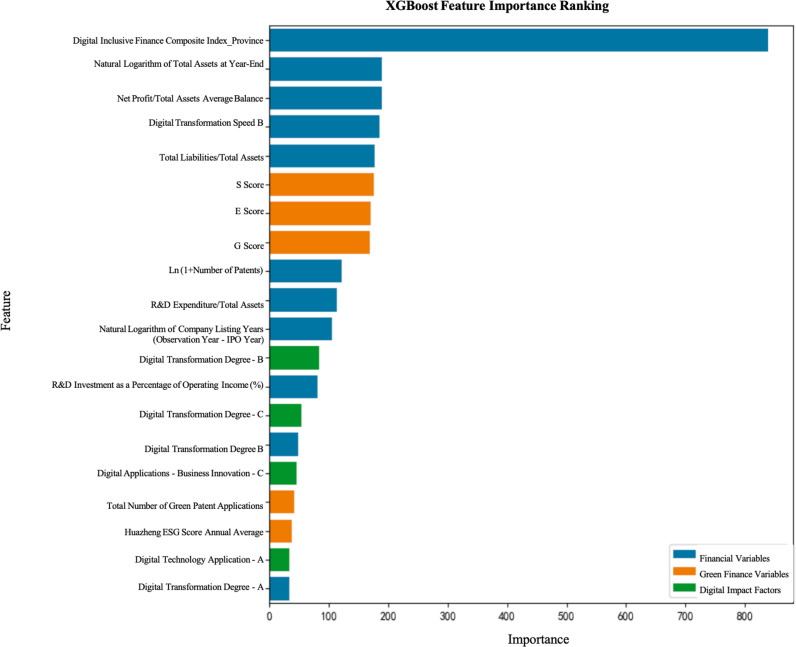
Feature Importance of the XGBoost Model.

According to the empirical analysis results, the Digital Inclusive Finance Comprehensive Index_Province has been identified as the most critical influencing factor across all models, further validating the core role of green finance in driving the digital transformation of the port and shipping industry. Green finance not only helps enhance the efficiency of resource allocation within the port and shipping sector but also promotes low-carbon development by directing funds towards sustainable projects. Additionally, financial indicators such as the natural logarithm of total assets at year-end and the average balance of net profit over total assets show significant impact in multiple models, indicating that financial health plays a crucial role in the operational efficiency and market competitiveness of port and shipping enterprises (See Figs 1 and 2).

### 5.4. Model residual analysis

**Fig 3:**
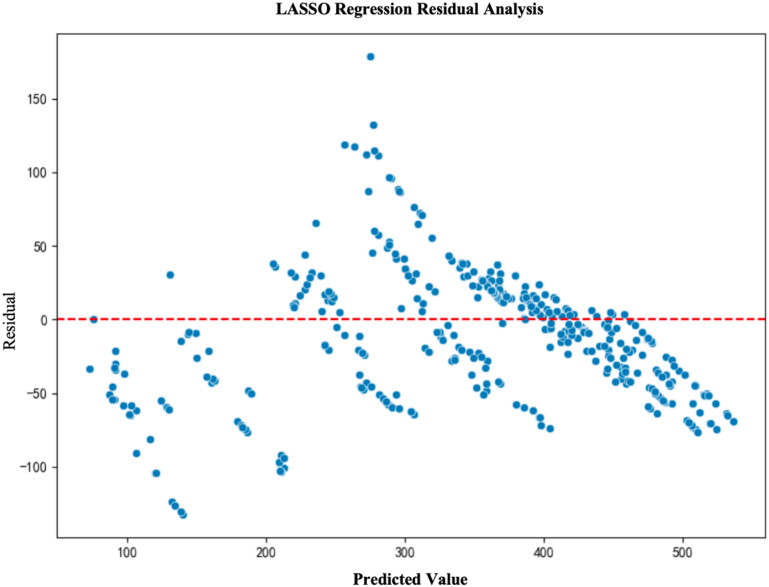
LASSO Regression Residual Analysis.

**Fig 4:**
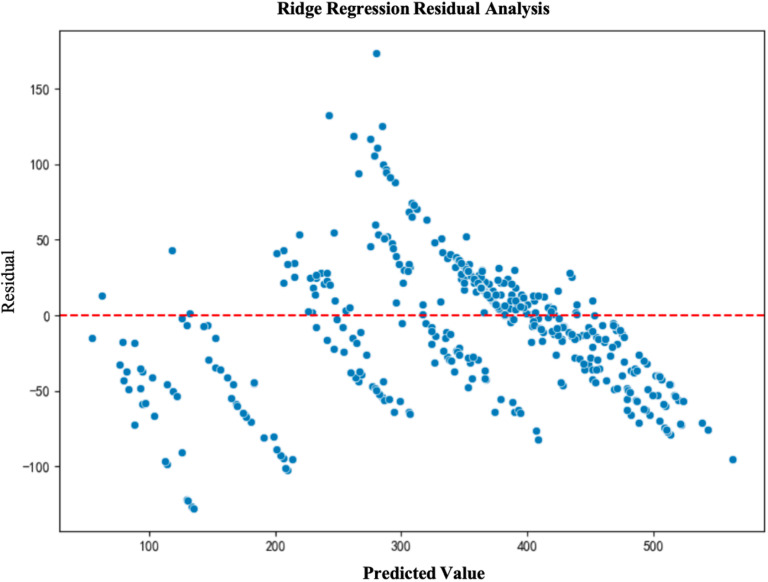
Ridge Regression Residual Analysis.

**Fig 5:**
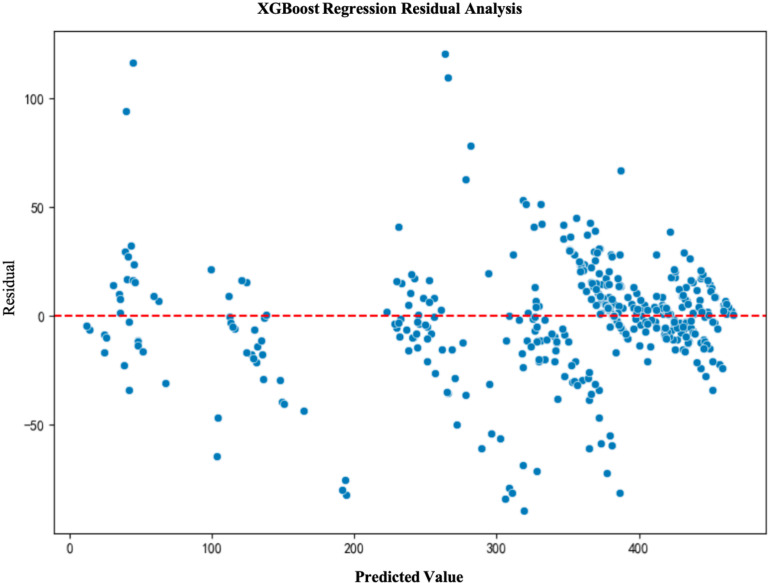
XGBoost Regression Residual Analysis.

**Fig 6:**
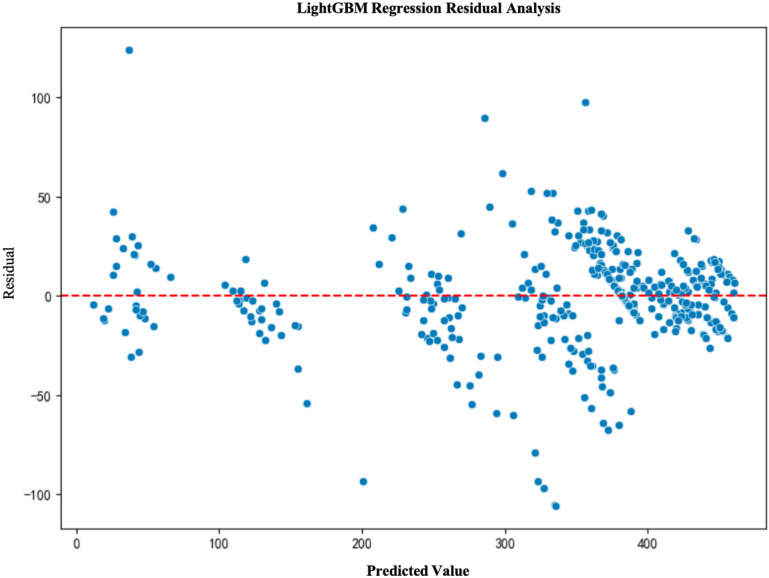
LightGBM Regression Residual Analysis.

To further validate the fitting performance and prediction accuracy of the models, we conducted a residual analysis for each model. Residual analysis helps to assess the distribution of prediction errors and determine whether the model exhibits any systematic bias. The results of the residual analysis show that the residuals of the LightGBM and XGBoost models are primarily concentrated around zero and are evenly distributed, indicating good model fit. In contrast, due to the inherent limitations of the multiple linear regression model, its residuals are unevenly distributed, leading to poor performance(See Figs 3–6).

## 6. Conclusion

This study analyzes the driving factors behind the digital transformation of Chinese port and shipping enterprises and draws the following conclusions:

First, the Digital Inclusive Finance Composite Index is identified as the most significant influencing factor across all models. A favorable green financial environment in certain provinces can provide financial support and policy incentives, reducing the costs and risks associated with digital transformation, thereby accelerating the transformation of port and shipping enterprises.

Second, the financial condition of port and shipping enterprises, including factors such as size, profitability, and financial stability, has a significant impact on digital transformation. Larger enterprises have more resources to invest in digital transformation, whereas high debt ratios and low profitability may limit investments in digital initiatives.

Third, the investment and progress in digital technology applications and the speed of transformation directly affect the degree of digital transformation. The maturity of digitalization and the level of technology application significantly boost the transformation of port and shipping enterprises.

Fourth, the performance of port and shipping enterprises in environmental, social, and governance (ESG) aspects promotes digital transformation. Enhancing the green image can help enterprises gain policy and financial support, thereby facilitating their digital transformation.

### 6.1. Improving green financial policies and optimizing the financing environment

Governments and financial institutions should actively promote the improvement of green financial policies, expand the Digital Inclusive Finance Composite Index, and establish low-cost, long-term financing channels while providing corresponding tax incentives and technical support. In light of the uneven economic development across regions, it is recommended to formulate differentiated green financial policies that encourage port and shipping enterprises in different regions to obtain targeted financial support based on their specific needs, thus reducing the risks and costs of digital transformation and green production. Furthermore, the government should enhance the transparency and credibility of green finance, guide the financial market to focus more on green innovation projects, and promote the deep integration of capital and green technologies, thereby providing more substantial funding guarantees for enterprise transformation.

### 6.2. Promoting financial health and stability to ensure sustainable development

Enterprises should optimize their financial structures and maintain an appropriate debt ratio to improve profitability and financial stability. For larger port and shipping enterprises, rational financial planning and prudent debt management will provide a more solid financial foundation for digital transformation. On this basis, enterprises should increase investment in research and development, especially in green and digital technologies, to avoid the adverse impact of excessive debt on sustainable development. At the same time, financial stability will help enterprises maintain sufficient risk resistance in the face of market fluctuations, thereby providing strong backing for the implementation of digital and green development strategies.

### 6.3. Increasing investment in technological innovation and digital applications to enhance enterprise competitiveness

Promoting technological innovation is the core driving force for enhancing the digital transformation of port and shipping enterprises. Enterprises should increase investments in the research and development of digital and green technologies to gradually improve their digital maturity and technology application levels. Moreover, it is essential to actively pursue green technology patents and encourage enterprises to achieve breakthroughs and industrialize green production technologies during the innovation process.

### 6.4. Research limitations and future outlook

Although this study provides important insights into the driving factors behind the digital transformation of Chinese port and shipping enterprises, there are several limitations. Firstly, the research sample comes from publicly available data, which may introduce sample selection bias. Additionally, this study primarily focuses on the key factors influencing the digital transformation of port and shipping enterprises. However, digital transformation is a complex, multi-dimensional process, and future research can explore additional dimensions of influencing factors and the relationships among these factors.
